# The Effects of Adipose-Derived Stem Cells in a Rat Model of Tobacco-Associated Erectile Dysfunction

**DOI:** 10.1371/journal.pone.0156725

**Published:** 2016-06-03

**Authors:** Yun-Ching Huang, Yi-Hung Kuo, Yan-Hua Huang, Chih-Shou Chen, Dong-Ru Ho, Chung-Sheng Shi

**Affiliations:** 1 Division of Urology, Department of Surgery, Chang Gung Memorial Hospital, Chiayi, Taiwan; 2 Graduate Institute of Clinical Medical Sciences, College of Medicine, Chang Gung University, Taoyuan, Taiwan; 3 Division of Colon and Rectal Surgery, Department of Surgery, Chang Gung Memorial Hospital, Chiayi, Taiwan; 4 Department of Occupational Therapy, School of Health and Human Services, College of Professional Studies, California State University, Dominguez Hills, Carson, CA, United States of America; Max-Delbrück Center for Molecular Medicine (MDC), GERMANY

## Abstract

Tobacco use is associated with erectile dysfunction (ED) via a number of mechanisms including vascular injury and oxidative stress in corporal tissue. Adipose derived stem cells (ADSC) have been shown to ameliorate vascular/corporal injury and oxidative stress by releasing cytokines, growth factors and antioxidants. We assessed the therapeutic effects of intracavernous injection of ADSC in a rat model of tobacco-associated ED. Thirty male rats were used in this study. Ten rats exposed to room air only served as negative controls. The remaining 20 rats were passively exposed to cigarette smoke (CS) for 12 weeks. At the 12-week time point, ADSC were isolated from paragonadal fat in all rats. Amongst the 20 CS exposed rats, 10 each were assigned to one of the two following conditions: (i) injection of phosphate buffered saline (PBS) into the corpora cavernosa (CS+PBS); or (ii) injection of autologous ADSC in PBS into the corpora cavernosa (CS+ADSC). Negative control animals received PBS injection into the corpora cavernosa (normal rats [NR] + PBS). After injections all rats were returned to their previous air versus CS exposure state. Twenty-eight days after injection, all rats were placed in a metabolic cage for 24-hour urine collection to be testing for markers of oxidative stress. After 24-hour urine collection all 30 rats also underwent erectile function testing via intracavernous pressure (ICP) testing and were then sacrificed. Corporal tissues were obtained for histological assessment and Western blotting. Mean body weight was significantly lower in CS-exposed rats than in control animals. Mean ICP, ICP /mean arterial pressure ratio, serum nitric oxide level were significantly lower in the CS+PBS group compared to the NR+PBS and CS+ADSC groups. Urine markers for oxidative stress were significantly higher in the CS+PBS group compared to the NR+PBS and CS+ADSC groups. Mean expression of corporal nNOS and histological markers for endothelial and smooth muscle cells was significantly lower, and tissue apoptotic index significantly higher, in the CS+PBS group compared to the NR+PBS and CS+ADSC groups. Our findings confirm that chronic tobacco exposure causes ultrastructural damage to the corporal tissue and increases systemic oxidative stress states. Treatment with ADSC ameliorates these adverse effects and holds promise as a potential therapy for tobacco-related ED.

## Introduction

Erectile dysfunction (ED) is defined as the inability to attain and/or maintain an erection sufficient for satisfactory sexual intercourse [1]. ED affects 29% men in Taiwan over the age of 40 years and will become more prevalent as the population ages [2]. Although ED is a benign disorder, it may cause substantial psychological problems and can have a significant negative impact on quality of life for men and their partners [3].

Several treatment options are available for ED. Oral phosphodiesterase type 5 inhibitors (PDE5I) are the first line pharmacotherapy of choice for men with ED who do not have a specific contraindication to their use [4]. However, PDE5I do not produce sufficient tumescence in all patients nor do they resolve the underlying functional disorder. Furthermore, while PDE5I are generally safe, a variety of mild but troublesome adverse effects including headache, flushing, dyspepsia, nasal congestion, abnormal vision, and back pain may reduce their suitability for some patients [5]. Alternative treatments such as intracavernous injection, vacuum tumescence devices, and penile prosthesis may be options for some men, but the tolerability of these agents is variable. A treatment that resolves the underlying disease process of ED would be preferable to currently available “on demand” interventions.

One of the therapeutic strategies currently being evaluated for definitive management of ED is stem cell therapy. Embryonic stem cells exhibit unlimited differentiation potential both *in vitro* and *in vivo* and are an ideal source for cell based therapies. However, there are substantial legal, ethical, and medical concerns regarding their use [6]. Somatic cells (e.g. mouse fibroblasts) have been transformed into pluripotent, stem-cell-like cells by introducing a set of embryonic transcription factors [7]. Unfortunately, the risk of malignant transformation is high in induced pluripotent stem cells [8]. Therefore, the greatest potential source for stem cell therapies appears to be progenitor cells isolated from adult tissues such as adipose. Adipose-derived stem cells (ADSC) are multipotent progenitor cells isolated from the stromal vascular fraction of adipose tissue [9]. Investigators have postulated a number of nonexclusive mechanisms (e.g. differentiation into somatic cells, secretion of cytokines and growth factors, reduction in oxidative stress) through which ADSC may restore tissue integrity in disease states [10, 11].

Tobacco use has been linked to significantly higher rates of ED with greater or longer duration of exposure leading to additive increases in risk [12]. The mechanisms of tobacco-associated ED are myriad and likely include oxidative stress and vascular injury [13, 14]. We previously reported impaired penile hemodynamics, decreased penile neuronal nitric oxide synthase (nNOS) and expression of endothelial and smooth muscle markers, and increased oxidative stress and apoptosis state in a rat model of ED from cigarette smoke (CS) exposure [15]. In this study, we investigate the efficacy of ADSC in the treatment of impaired penile hemodynamics and penile histological changes in the same rat model of CS-related ED.

## Materials and Methods

### Experimental design

This study was carried out in strict accordance with the rules of Association for Assessment and Accreditation of Laboratory Animal Care International and was approved by the Institutional Animal Care and Use Committee of the Laboratory Animal Center at our institution. All surgery was performed under Zoletil-50 anesthesia, and efforts were made to minimize discomfort in subject animals.

Thirty 12-week old male Sprague-Dawley rats were obtained from BioLASCO Taiwan Co., Ltd and divided into 3 groups. Ten rats exposed only to room air served as negative controls. The remaining 20 rats were passively exposed to CS for 12 weeks. This has been shown in a prior study to induce impairment of penile hemodynamics in rats [15]. Twelve weeks after initiation of room air or CS exposure, all 30 rats underwent para-gonadal fat harvest to procure ADSC. The 20 CS-exposed rats were randomly divided into 2 groups: (i) injection of phosphate buffered saline (PBS) into the corpora cavernosa (CS+PBS); and (ii) injection of 1 million autologous ADSC into the corpora cavernosa (CS+ADSC). Negative control animals received PBS injection into the corpora cavernosa (normal rats [NR] + PBS). After injections all rats were returned to their previous air versus CS exposure state. Twenty-eight days after injection, all rats were placed in a metabolic cage for 24-hour urine collection. Urine samples were obtained to measure systemic oxidants. After urine collection, all 30 rats also underwent penile hemodynamic testing and were then sacrificed. Serum samples were obtained to measure serum testosterone, nitric oxide (NO) and antioxidant scavenging enzymes. Penile tissues were collected for Masson’s trichrome, immunohistochemistry, and Western blot analyses.

### Cigarette smoke exposure

The procedures were performed as described previously [15]. Briefly, rats were housed in enclosed plastic cages measuring 28×28×19 cm. Rats exposed to CS were housed in cages treated with a constant influx of CS using a small air pump. One lighted cigarette was placed into an inverted tube connected to a peristaltic pump communicating with the cage. The smoke was distributed by a small fan and exited through a second opening. Cigarettes (contents: 10 mg of tar and 0.8 mg of nicotine, Marlboro, Philip Morris, Richmond, VA, USA) with filter was combusted consecutively to exhaustion at a rate of one cigarette per 6 minute. Rats in the CS groups were exposed to 2 hours of continuous CS per day, 5 days per week.

### ADSC harvest, processing and EdU labeling

ADSC was isolated as previously described [16]. Briefly, under Zoletil-50 anesthesia, perigonadal adipose tissue was harvested in all rats. The adipose specimen was washed with sterile PBS and minced, followed by digestion with 0.075% collagenase I (Sigma-Aldrich, St. Louis, MO, USA) for 1 hour at 37C with shaking. The top lipid layer was removed and the remaining liquid portion was centrifuged at 220 X gravity for 10 minutes. The pellet was treated with 160 mM NH_4_Cl for 10 minutes to lyse red blood cells. Remaining cells were suspended in culture medium and placed in a culture dish. The culture dishes were stored in a 5% CO_2_ incubator for 3 to 5 days to allow ADSC colonies to form. ADSC were then cultured and labeled with 5-ethynyl-2’-deoxyuridine (EdU, Invitrogen, Carlsbad, CA, USA) for 48 hours before injection.

### ADSC Injection

As described in detail previously [16], under Zoletil 50 (20 mg/kg, IM) anesthesia, a skin incision was made to expose the penis. Using blunt dissection, the penile base and crura were exposed. The corpora cavernosa were then gently cannulated using a 25-gauge needle. The penile base was constricted with a polyethylene (PE)-90 tourniquet to reduce the potential of cells to escape into systemic circulation. Each rat underwent injection of either 0.3mL PBS (NR+PBS and CS+PBS groups) or 1 million autologous ADSC in 0.3 mL PBS into the corpora cavernosa (CS+ADSC group). Following injection, the needle and tourniquet were left in place for 5 minutes. The needle and tourniquet were then removed and the wound closed in one layer with absorbable suture.

### Erectile Function Evaluation

At 28 days post-injection, erectile function testing was done as prior study [15]. Under anesthesia with Zoletil 50 (20 mg/kg), the bilateral cavernous nerves were isolated via midline laparotomy. The penis was cannulated with a heparinized 23-gauge needle connected to a real-time continuous pressure transducer. The cavernous nerves were then stimulated with a stainless steel bipolar hook electrode; stimulation parameters were 50 seconds continuous trains at 20 Hz, and 1.5 mAmp (A-M Systems, Sequim, WA, USA) [17]. Real-time response of the erectile tissue was determined by a change in intracavernous pressure (ICP) using LabView 6.0 software (National Instruments, Austin, TX, USA). The maximum increase in ICP for three stimuli per side for each animal was utilized for further analysis. After functional testing, systemic blood pressure was measured via aortic cannulation. Mean arterial pressure (MAP) was calculated by the formula MAP = (2/3 diastolic blood pressure + 1/3 systolic blood pressure). Penile hemodynamics are reported as the ICP/MAP ratios. After aortic puncture, serum was obtained from the aortic cannula and the corpora cavernosa were harvested for histological and molecular studies. The animals were then euthanized by bilateral thoracotomy [16].

### Oxidative Stress Assay

In serum, we analyzed antioxidant scavenging enzymes, including plasma total antioxidant capacity (TAC, as assessment of cumulative action of all the antioxidants present in plasma and body fluids) and erythrocyte glutathione peroxidase (GPx, a physiologic free radical scavenger) [18, 19]. The plasma TAC and erythrocyte GPx were determined using ferric-reducing ability of plasma assay (Roche Diagnostics, Mannheim, Germany) and the colorimetric kinetic method (Randox Laboratories, Co. Antrim, UK), respectively, according to the manufacturer’s recommendation [20, 21].

We evaluated the oxidative stress state in urine, including malondialdehyde (MDA, the most commonly used marker for lipid peroxidation) and 8-hydrox-2’-deoxyguanosine (8-OHdG, one of the predominant forms of free radical-induced oxidative DNA damage products) [22, 23]. The concentration of MDA and 8-OHdG were measured by the thiobarbituric acid method (Oxford Biomedical Research, Rochester Hills, MN, USA) and enzyme-linked immunosorbent assay (Trevigen, Gaithersburg, MD, USA), respectively, according to the manufacturer ‘s instructions [24]. The urinary concentration of MDA and 8-OHdG were normalized to urinary creatinine to mitigate differences potentially attributable to urine volume.

### Griess nitrite assay

NO production after erectile function testing was estimated spectrophotometrically as a formed nitrite, which is one of two primary, stable and nonvolatile breakdown productions of NO. To measure serum nitrite content, 50μL of the nitrite standards or serum was incubated with 50 μL of Griess reagent for 5–10 minutes, and then *N*-1-napthylethylenediamine dihydrochloride solution for another 5–10 minutes (Promega, WI, USA). Absorbance was measured at 550 nm using a spectrophotometric reader. The nitrite content was calculated based on a standard curve and expressed as μM [25].

### Western Blot Analysis

As described in detail previously, the urethra was dissected free from penile tissue samples [15]. Corporal tissue from the penile base were homogenized in tissue protein extraction exigent (Thermo Fisher Scientific Inc., Waltham, MA, USA). The lysate was centrifuged at 17,900 g for 15 minutes, and the supernatant was collected as the protein samples. Tissue lysates containing 40 μg of protein were electrophoresed in gradient SDS-PAGE and then transferred onto a PVDF membrane (Millipore, Darmstadt, Germany). Detection of protein on the membrane was performed with the ECL kit (Amersham Life Sciences, Pittsburgh, PA, USA) using a rabbit anti-nNOS (Santa Cruz Biotechnology, Dallas, TX, USA). Before re-probing with an anti-β-actin antibody, the membrane was stripped in 62.5mM Tris-HCl, pH 6.7, 2% SDS, 10 mM 2-mercaptoethanol at 56°C for 30 minutes and then washed four times in 1xTBST. Results were quantified by densitometry.

### Immunohistochemical Staining

Penile mid-shaft tissues were harvested, fixed and further processed for immunohistochemical staining as previously described [15]. Briefly, tissues were fixed in cold 2% formaldehyde and 0.002% picric acid in 0.1 M phosphate buffer, pH 8.0, for 4 hours followed by overnight immersion in buffer containing 30% sucrose. The specimens were then embedded in OCT Compound (American Master Tech Scientific, Lodi, CA, USA) and stored at -70°C until use. Sections were cut at 5 μm, mounted into charged slides and air dried for 5 minutes. Representative slides were stained with Masson’s trichrome for connective tissue and smooth muscle histology.

For immunohistochemical examination, tissue sections were stained with mouse anti-rat endothelial cell antigen-1 (RECA-1, Abcam Inc, Cambridge, MA, USA) and terminal deoxynucleotidyl transferasemediated deoxyuridine triphosphate nick-end labeling (TUNEL, Roche Diagnostics Corporation, Indianapolis, IN, USA) using standard techniques [16]. To visualize ADSC, EdU staining with immunostaining for α-smooth muscle actin (α-SMA) was done with mouse anti-α-SMA (Sigma-Aldrich, St. Louis, MO, USA) and freshly made Click-IT reaction cocktail (Invitrogen, Carlsbad, CA, USA). Nuclear staining was performed with 4’,6-diamidino-2-phenylindole (DAPI, Invitrogen, Carlsbad, CA, USA) [26].

### Image and Statistical Analysis

The image results were analyzed by computerized densitometry using Image-Pro plus imaging software (Media Cybernetics, Silver Spring, MD, USA) coupled to a digital camera (Nikon DXM1200) and ACT-1 software (Nikon Instruments Inc., Melville, NY, USA). Data were analyzed with Prism version 6 (GraphPad Software, La Jolla, CA, USA) and expressed as mean ± standard error of the mean for all continuous variables. Continuous data were compared using one-way analysis of variance; followed by the Tukey–Kramer test for post hoc comparisons. Statistical significance was set at *P* < 0.05.

Quantification of Masson’s trichrome and RECA-1 was performed at x40 magnification (comprising approximately one half of one corporal body). To quantify Masson’s trichrome staining, the specimen was assessed for smooth muscle (stained in red) and collagen (stained in blue); data are expressed as the percentage of smooth muscle normalized to collagen content. For RECA-1 staining, the area of positive staining was assessed and results expressed as the percentage of positive area vs. total area of the corpora cavernosa. For TUNEL staining, five randomly selected fields of the corpora cavernosa were examined at x100 magnification; results were expressed as the number of TUNEL-positive nuclei in the corpora cavernosa. All histological analyses were performed by one reviewer who was blinded to treatment group.

## Results

### Body Weight, Erectile Function, Serum NO and Testosterone

Total body weight was significantly lower in CS+PBS and CS+ADSC groups than in NR+PBS group (*p* = 0.0018, Table 1). There was no statistically significant difference in weight between the CS+PBS and CS+ADSC groups (*p* = 0.9988).

**Table 1:** Total body weight, erectile function and serum biochemistry.

Groups	NR+PBS	CS+PBS	CS+ADSC	*P* value
Body weight (gram)	608 ± 24.4*	534 ± 4.6	535 ± 7.4	0.0018
ICP (mm-Hg)	88±7.1	54±10.8*	85±3.8	0.0105
MAP (mm-Hg)	106±6.4	116±3.7	119±6.4	0.2577
ICP/MAP ratios	0.83 ± 0.020	0.46 ± 0.079*	0.69 ± 0.041	0.0011
Testosterone (ng/mL)	4.3 ± 0.67	3.1 ± 0.46	4.3 ± 1.06	0.4799
Nitric oxide (μM)	3.2 ± 0.31	2.0 ± 0.08*	2.9 ± 0.21	0.0011

Eight to ten rats were selected for analysis from each group.

* Versus other groups *p* < 0.05.

Abbreviations: NR, normal rat; PBS, phosphate buffered saline; CS, cigarette smoking; ADSC, adipose-derived stem cells; ICP, intracavernous pressure; MAP, mean arterial pressure.

Mean ICP and ICP/MAP ratios were significantly lower in CS+PBS group compared to NR+PBS and CS+ADSC groups (*p* = 0.0105 and *p* = 0.0011, respectively). The CS+ADSC group also had lower mean ICP and ICP/MAP ratio than the NR+PBS group but the difference did not reach statistical significance (*p* = 0.9191 and *p* = 0.2403, respectively). Although there was no statistically significant difference in MAP (*p* = 0.2577), there was a trend of higher MAP in CS-exposed rats than in control animals.

Serum NO was significantly higher in NR+PBS and CS+ADSC groups than in CS+PBS group (*p* = 0.0011). Although there was no statistically significant difference in serum testosterone (*p* = 0.4799), there was a trend towards decreased testosterone level in the rats that exposed to CS without ADSC treatment (Table 1).

### Oxidative Stress State

TAC level in the CS+PBS group was significantly lower relative to all other groups (*p* = 0.0191) but there was no significant difference between NR+PBS and CS+ADSC groups (*p* = 0.9943). The level of 8-OHdG in the CS+PBS group was significantly higher than what was observed in NR+PBS and CS+ADSC groups (*p* = 0.0202); no significant differences between NR+PBS and CS+ADSC were noted for 8-OHdG (*p* = 0.9798). There were no significant differences in GPx or MDA levels between the three groups (Table 2).

**Table 2:** Oxidative and anti-oxidative activity in plasma and urine.

Groups	NR+PBS	CS+PBS	CS+ADSC	*P* value
TAC (μmol/L)	217 ± 13.2	140 ± 9.6*	220 ± 30.3	0.0191
GPx (U/g)	828 ± 119.2	592 ± 59.2	752 ± 69.4	0.2105
8-OHdG/creatinine (ng/mg)	34.2 ± 2.30	49.1 ± 5.07*	35.2 ± 3.72	0.0202
MDA/creatinine (μmol/g)	9.8 ± 1.70	11.4 ± 1.33	9.7 ± 2.34	0.7489

Eight to ten rats were selected for analysis from each group.

To avoid the effect of urine volume fluctuation, the urinary concentration of 8-OHdG and MDA were normalized to urinary creatinine.

The antioxidant scavenging enzymes, including TAC and GSH, were determined in plasma and erythrocyte, respectively.

* Versus other groups *p* < 0.05.

Abbreviations: NR, normal rat; PBS, phosphate buffered saline; CS, cigarette smoking; ADSC, adipose-derived stem cells; TAC, total anti-oxidant capacity; GPx, glutathione peroxidase; 8-OHdG, 8-hydrox-2’-deoxyguanosine; MDA, malondialdehyde.

### Tracking Transplanted ADSC

**Fig 1:**
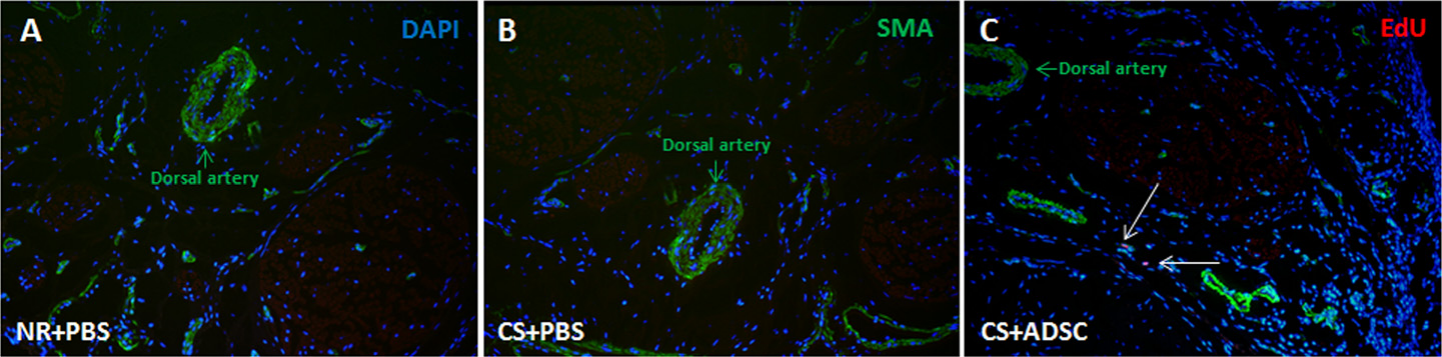
Tracking transplanted ADSC. Penis was stained with DAPI (blue area), anti-α-SMA antibody (green areas) and Alexa-594 (red areas). Only EdU positive cells showed red fluorescence (arrows). No EdU positive cell was identified in NR+PBS (A) and CS+PBS (B) groups. Only few EdU positive cells were identified in the dorsal penis of CS+ADSC group (C). Original magnification: x 400. Abbreviations: NR, normal rat; CS, cigarette smoke; PBS, phosphate buffered saline; ADSC, adipose-derived stem cell; DAPI, 4’,6-diamidino-2-phenylindole; SMA, smooth muscle actin; EdU, 5-ethynyl-2’-deoxyuridine.

Autologous EdU labeled ADSC were transplanted into corpora cavernosa (CS+ADSC group) and identified by Alexa-594 stain. Four weeks after intracavernous injection, histological examination revealed no EdU-labeled cells in the corpora cavernosa but scant cells in the dorsal penis, suggesting that ADSC either migrated or died rather than differentiating into somatic cells (Fig 1).

### nNOS Western Blot

**Fig 2:**
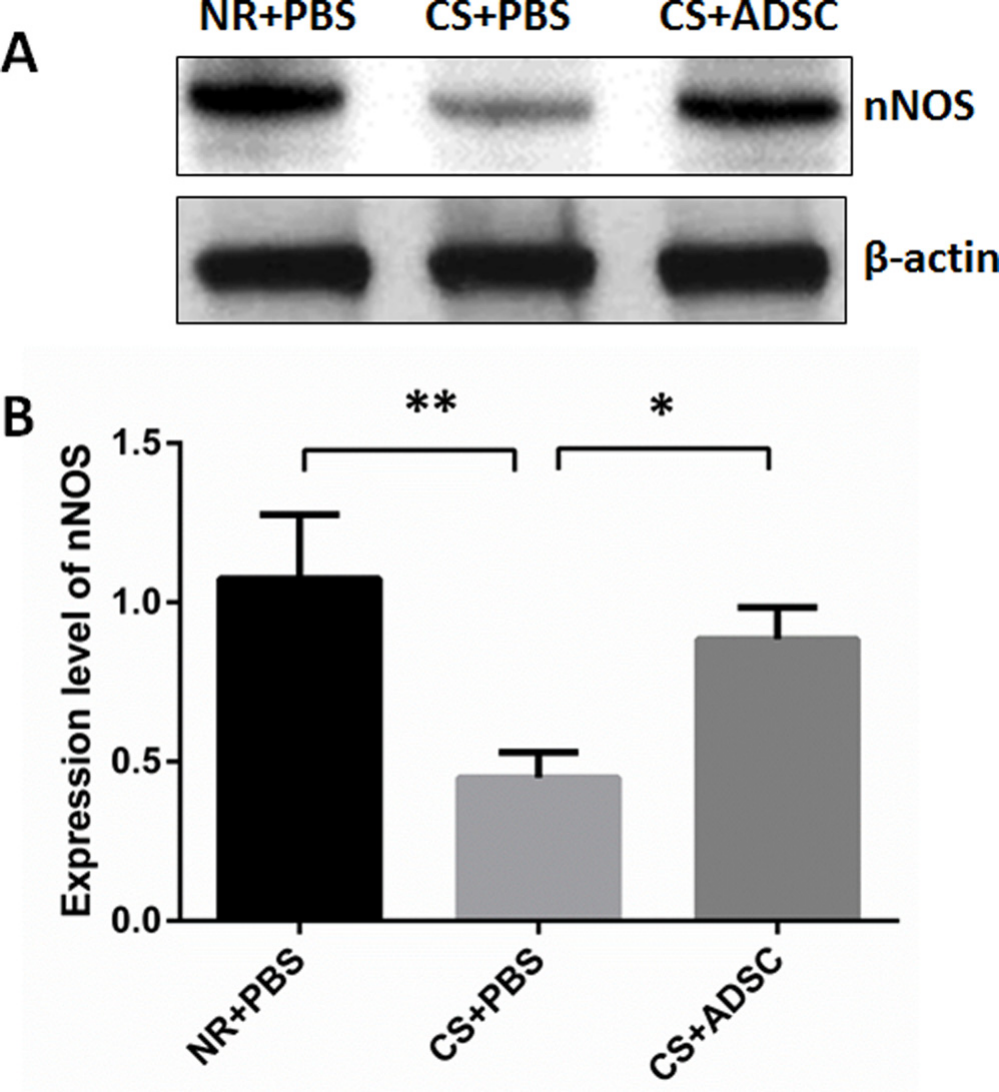
Western blot for nNOS. (A) Western blot normalized to β-actin; (B) mean nNOS positivity. Eight to ten rats were selected for nNOS analysis from each group. Mean nNOS was significantly lower in the CS+PBS group compared with all others. *, *p* < 0.05; **, *p* < 0.01. Abbreviations: nNOS, neuronal nitric oxide synthase; NR, normal rat; PBS, phosphate buffered saline; CS, cigarette smoke; ADSC, adipose derived stem cell.

Protein expression of penile nNOS was significantly lower in the CS+PBS group (0.45±0.080) compared with NR+PBS (1.07±0.201) and CS+ADSC (0.88±0.099) groups (*p* = 0.0065). There was no significant difference in nNOS expression between the NR+PBS and CS+ADSC groups (*p* = 0.6107). Representative images of nNOS Western blot are presented in Fig 2.

### Endothelial Integrity

**Fig 3:**
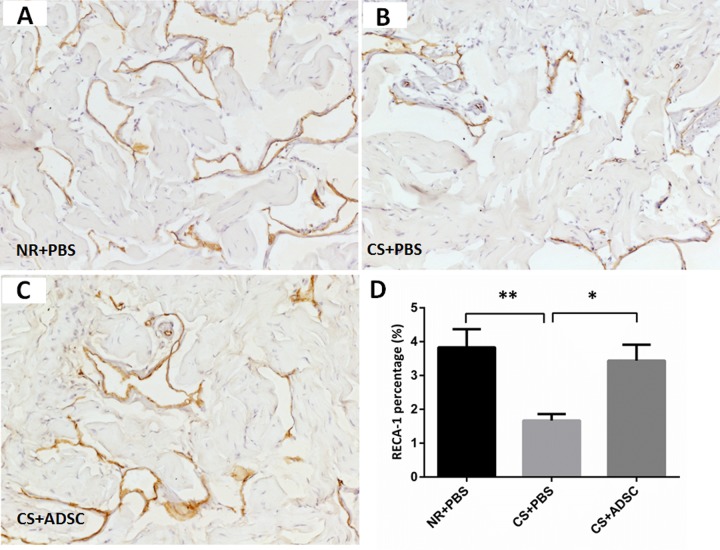
Endothelium content in the corpora cavernosa. Representative images from rats in the NR+PBS (A), CS+PBS (B) and CS+ADSC (C) groups. Endothelial cells are stained brown with the RECA-1 antibody. Eight to ten rats were selected for endothelium content analysis from each group. The intense positivity of RECA-1 was lower in CS+PBS than in NR+PBS and CS+ADSC groups (D). Magnification is x200. *, *p* < 0.05; **, *p* < 0.01. Abbreviations: NR, normal rat; PBS, phosphate buffered saline; CS, cigarette smoke; ADSC, adipose derived stem cell; RECA-1, rat endothelial cell antigen-1.

RECA-1 staining for endothelial tissue in the corpora cavernosa was significantly lower in the CS+PBS (1.7±0.19) group relative to the NR+PBS (3.8±0.54) and CS+ADSC (3.4±0.47) groups (*p* = 0.0055). There was no significant difference in RECA-1 positivity between NR+PBS and CS+ADSC groups (*p* = 0.8146). Representative images of RECA-1 staining are presented in Fig 3.

### Smooth Muscle Content

**Fig 4:**
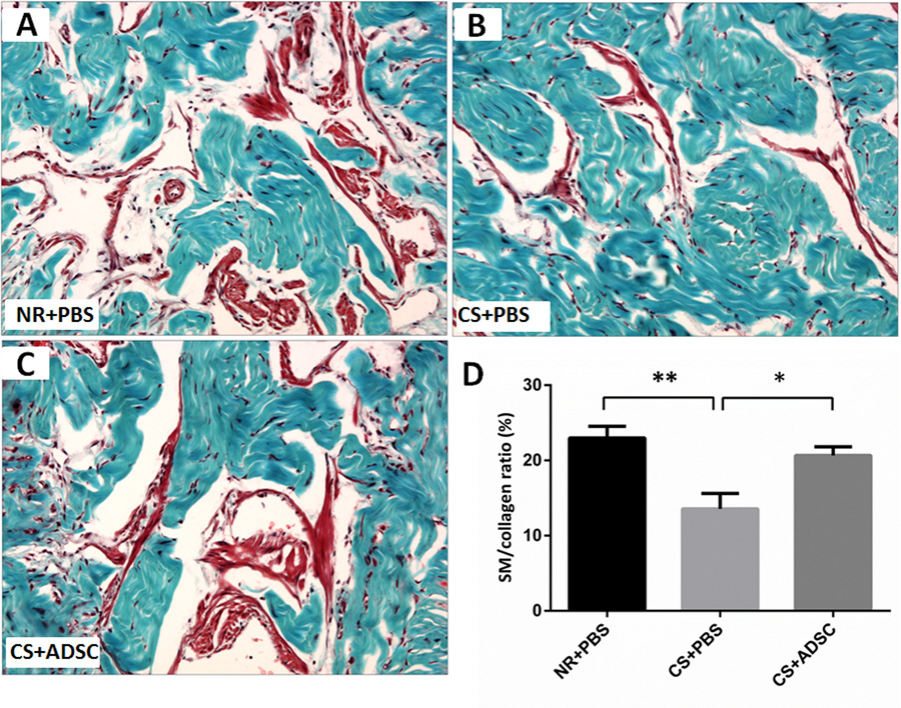
Smooth muscle content in the corpora cavernosa. Representative images from rats in the NR+PBS (A), CS+PBS (B) and CS+ADSC (C) groups were shown. Smooth muscle and connective tissue are stained with red and blue, respectively, using the trichrome method. Eight to ten rats were selected for smooth muscle content analysis from each group. The smooth muscle content was lower in CS+PBS group than in NR+PBS and CS+ADSC groups (D). Magnification is x200. *, *p* < 0.05; **, *p* < 0.01. Abbreviations: NR, normal rat; PBS, phosphate buffered saline; CS, cigarette smoke; ADSC, adipose derived stem cell.

Masson’s trichrome demonstrated a significantly lower smooth muscle to collagen percentage in CS+PBS (13.6±2.04) group compared to the NR+PBS (23.0±1.55) and CS+ADSC (20.7±1.17) groups (*p* = 0.0039). There was no significant difference in smooth muscle to collagen ration between NR+PBS and CS+ADSC groups (*p* = 0.7104). Representative images of Masson’s trichrome staining are presented in Fig 4.

### TUNEL Immunohistochemistry

**Fig 5:**
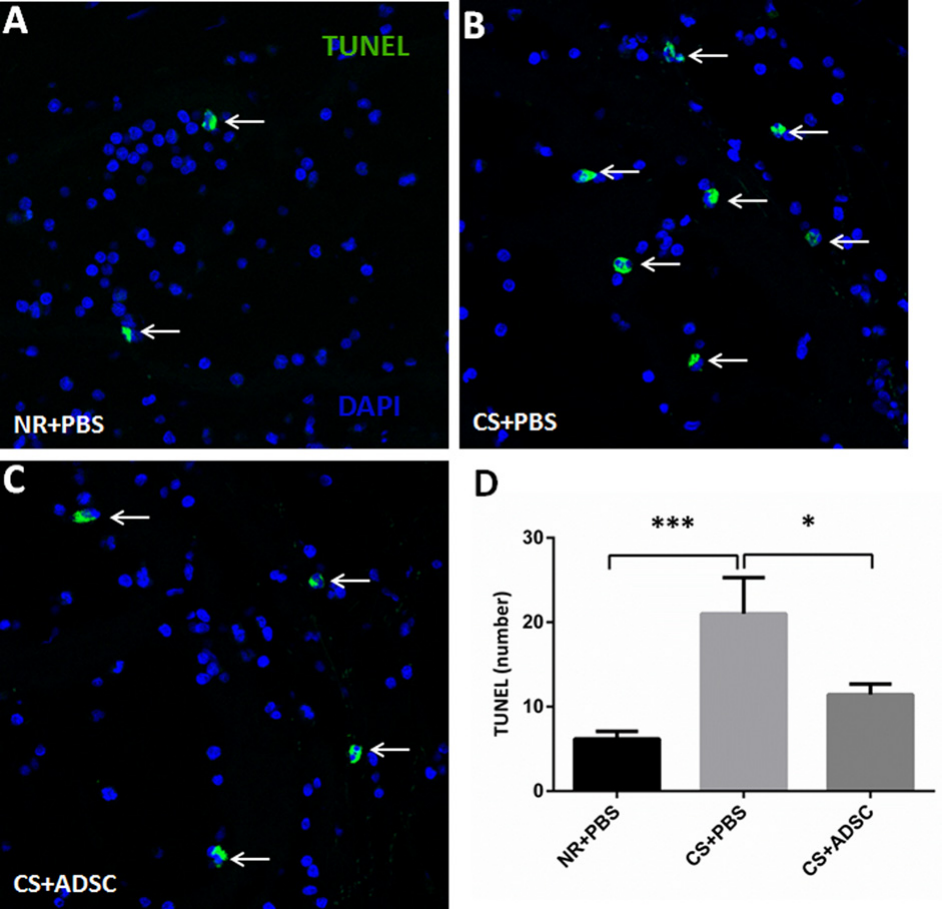
Analysis of apoptosis-positive cells by TUNEL stain. Representative images from rats in the NR+PBS (A), CS+PBS (B) and CS+ADSC (C) groups were shown. The corpora cavernosa was stained with TUNEL stain (green) and DAPI (blue) for the visualization of apoptotic cells and cell nuclei, respectively. Nine to ten rats were selected for apoptosis analysis from each group. The result showed the increase of the number of apoptosis-positive cells in CS+PBS group than in NR+PBS and CS+ADSC groups (D). Magnification is x 800. *, *p* < 0.05; ***, *p* < 0.001. Abbreviations: TUNEL, terminal deoxynucleotidyl transferasemediated deoxyuridine triphosphate nick-end labeling; NR, normal rat; PBS, phosphate buffered saline; CS, cigarette smoke; ADSC, adipose derived stem cell; DAPI, 4’,6-diamidino-2-phenylindole.

TUNEL-positive cells were significantly more frequent in CS+PBS (21.0±4.28) compared to the NR+PBS (6.2±0.89) and CS+ADSC (11.4±1.24) groups (*p* = 0.0013). There was no significant difference in the number of TUNEL positive cells between NR+PBS and CS+ADSC groups (*p* = 0.3207). Representative images of TUNEL staining are presented in Fig 5.

## Discussion

Epidemiological studies have clearly documented that cigarette smoke is associated with development of ED [27, 28]. Ethical considerations preclude the possibility of a randomized trial of tobacco as a cause of ED and hence observational data must suffice to establish a relationship between tobacco and ED. In this study, a rat model of tobacco-exposure related ED was used to investigate the therapeutic impact of ADSC. Tobacco exposed rats develop a phenotype consistent with tobacco-related ED in humans. CS exposure was associated with alternations in systemic redox state, increased penile apoptosis, and ultra-structural damage to the corporal nerve, smooth muscle, and endothelium. These effects likely mediate the impairment of penile hemodynamics observed in these rats. Lastly, transplanted ADSC may ameliorate tobacco-related ED, possibly via anti-oxidative effects. To our knowledge, this is the first study to demonstrate that the significant deleterious effects of CS exposure on physiology, penile hemodynamics, systemic redox status, endothelial, nNOS and smooth muscle contents may be reduced by ADSC.

The mechanism by which ADSC exerts a protective/restorative effect is unclear [29]. Indeed, in the face of a remarkable protective and regenerative effect, the engraftment of EdU-labeled ADSC was quantitatively very low in the corporal tissue 4 weeks after injection. The absence of significant cells incorporation into smooth muscle cells argues against differentiation into new cells as a major mechanism of ADSC. Based on our observations it is logical to hypothesize that paracrine release of cytokines, growth factors, and/or antioxidants by the transplanted ADSC is responsible for the observed effects. Further study is needed to determine which factor or combination of factors is driving the effects of ADSC *in vivo*.

Bonafede et al. demonstrated that ADSC can secrete exosomes to protect cells from oxidative damage *in vitro* [30]. Similarly, Pan et al. used a rat model of non-alcoholic fatty liver disease to demonstrate a significant reduction in oxidative stress and attenuation of disease progression in ADSC-treated animals [31]. In the present study, we confirmed that ADSC-treated animals had higher level of antioxidant scavenging enzymes (as determined by TAC expression in plasma) when comparing to control animals; this may underlie the efficacy of ADSC in treatment of smoking-associated compromise of penile hemodynamics in this model. Further study of the specific antioxidant enzymes secreted by ADSC in vivo is required to clarify this issue.

NO, a vasodilator produced by the endothelium and nerve terminals in the penis, is thought to be the principal stimulator of cavernosal relaxation and penile erection [32]. NO formation occurs via the regulatory actions of NOS in nitrergic nerves and penile endothelial cells. Although the pathophysiology of CS-induced ED is not entirely clear, studies from several laboratories and clinical trials have demonstrated that smoking-related ED is associated with reduced bioavailability of NO due to increased ROS, thereby resulting in oxidative stress [33]. Free radicals not only cause ultrastructural damage to the corporal tissue but also produce alteration in NOS activities in mice, rats and humans; all of these mechanisms are considered to play a role in chronic smoking-induced ED [15, 33, 34]. In the present study, low level of NO in plasma was observed in CS-exposed rats. CS-exposed rats also showed reduction of nNOS, endothelial and smooth muscle contents in corporal tissue. These results are consistent with previous findings in CS rats [15, 33]. Unique to this study, we have demonstrated that ADSC ameliorate these negative effects. We hypothesize from these findings that antioxidant therapy (and possibly stem cell therapy) may be of benefit for the prevention or treatment of ED in men who smoke. The validity and clinical application of these antioxidants in ED requires additional study.

An interesting finding of our study was that body weight was significantly lower in the CS-exposed rats than in the controls. This finding supports prior reports of decrease body weight in CS exposed rats [35]. Nicotine, the main addictive component of tobacco, is a well-known appetite suppressants and increases resting metabolic rate [36]. It is logical to conjecture that this effect may explain the decreased body weight in our model system.

The principal limitations of our study were a lack of molecular data on the underlying mechanisms of antioxidant effect and use of an animal model system. In fact, it is not clearly that the improvement of cavernosal structures is directly induced by ADSC implantation or is a consequence of a generalized effect of ADSC in cardiovascular system. We did not include a study arm that involved cessation of CS exposure, with or without ADSC injection. The nature of exposure (2 hours a day, five days a week) does not reflect the standard human exposure of repeated short doses throughout the course of the day. Differences in tobacco products may also contribute to variations in exposure that might change biological effects. These important questions will be the subject of additional research projects. Another critical question is the long-term durability of our results. The rats were followed for only 4 weeks. A longer term study would permit more longitudinal data on the durability of ADSC response in this animal model. Further research is now ongoing to determine the optimal protocol for cellular therapy of smoking-associated ED.

Nevertheless, this study is the first to use ADSC treatments in a rat model of tobacco associated ED. The findings suggest that intracavernous injection of ADSC holds promise as a therapeutic approach for the treatment of ED in men who smoke. Although this is a promising development, responsible physicians must continue to advise patients to stop using tobacco altogether as a critical health intervention that has implications even beyond sexual function.

## Conclusions

The current study further confirms that chronic CS exposure causes ultrastructural damage to the corporal tissue and alters systemic redox states as indicated by increase oxidative markers in the urine and decreased antioxidative markers in plasma. Intracavernous injection of autologous ADSC results in partial recovery of penile hemodynamics in a rat model of smoking associated ED. While this *in vivo* model demonstrate the benefits of ADSC, more comprehensive cellular and molecular analysis are needed to elucidate the true mechanism behind ADSC-mediated reversal of CS-exposure-induced ED.
